# Predictive features analysis and nomogram construction for predicting depression in elderly patients

**DOI:** 10.3389/fpsyg.2025.1628719

**Published:** 2025-08-11

**Authors:** Wei Lin, Zijun Zhao, Yingshan Yu, Hongbin Chen

**Affiliations:** ^1^Department of Geriatrics, Fuzhou First General Hospital Affiliated with Fujian Medical University, Fuzhou, Fujian, China; ^2^Department of Neurology, Fujian Medical University Union Hospital, Fuzhou, Fujian, China

**Keywords:** depression, predictive features, nomogram, elderly patients, depression screening model

## Abstract

**Introduction:**

In elderly populations, depression is highly prevalent among those with chronic diseases and cognitive impairment, leading to distress, disability, and poor medical outcomes. With the aging of the population, the prevalence of geriatric depression is rising rapidly. The Comprehensive Geriatric Assessment (CGA), a multidimensional approach, evaluates medical, psychological, and functional capacities to identify highrisk individuals and may be correlated with depression in the elderly.

**Methods:**

From 2021 to 2023, a total of 219 geriatric patients were recruited. These patients were divided into two groups: a modeling group of 153 patients and a validation group of 66 patients. We collected patients’ basic information and CGA results and analyzed them using univariate and multivariate regression. Independent variables influencing depression were identified.

**Results:**

Multivariate regression analyses revealed that several factors had an impact on depression in these patients, including social support level (SSRS), Pain, Anxiety, Basic Activities of Daily Living (BADL) and Gender. By integrating these factors into the nomogram, we found good predictive performance in the training set (AUC 0.867, 95% CI: 0.799–0.936) and in the test set (AUC 0.724, 95%CI:0.5919–0.894). The calibration and discrimination accuracy of the nomograms for predicting depression risk in the elderly were satisfactory, and the decision curve analysis demonstrated significant clinical utility.

**Discussion:**

The model demonstrated robust performance in our study and may constitute a valuable tool for clinical screening.

## 1 Introduction

Depression is a common and serious mental disorder. It has a high prevalence among the older population and profoundly impacts their lives. Global epidemiological data show that the prevalence of depression among community-dwelling older persons is approximately 10–15% (Gundersen and Bensadon, 2023). This prevalence can be as high as 30–40% among older persons with chronic illnesses or those in need of long-term care (Borges et al., 2021; Li et al., 2022). The disease not only impairs patients’ emotional wellbeing and quality of life, but also significantly increases the likelihood of cognitive decline, physical illness worsening, suicide risk, and premature death (Wang et al., 2025; Dai et al., 2024). As the global population ages, the prevalence of depression in the elderly is expected to rise further. It constitutes a growing public health burden due to increased functional dependence, dramatic increases in healthcare resource consumption, and heavy caregiver burden (Loyal et al., 2025; Dafsari et al., 2023). With the acceleration of global aging, exploring effective strategies for early identification and accurate prediction of geriatric depression has become a key breakthrough for improving health outcomes and optimizing healthcare resource allocation in older adults (Schutz et al., 2022).

Geriatric depression is a complex disorder characterized by multiple biological, psychological, and social factors. Studies have shown that reduced levels of brain-derived neurotrophic factor (BDNF) are associated with depressive symptoms. Additionally, some inflammatory factors, such as interleukin-6 (IL-6) and tumor necrosis factor-alpha (TNF-α), may be associated with depression and cognitive decline (Borges et al., 2022; Xue et al., 2025). Furthermore, studies have shown that depression in the elderly results from the dynamic interaction of multiple factors such as quality of life, emotional health, social engagement, and physical health. These factors form a complex ‘biopsychosocial’ etiological network (Han et al., 2023; Wang et al., 2020).

Studies have shown that active and healthy lifestyle factors can significantly buffer the risk of depression (Linnan et al., 2020): a strong social network provides emotional support and a sense of belonging; regular social activities and physical exercise play an antidepressant role by enhancing neuroplasticity and self-worth (Gao et al., 2024); and maintaining Activities of Daily Living (ADL) and Instrumental Activities of Daily Living (IADL) helps reduce depression risk and lowers the likelihood of developing depression (Cole et al., 2023). Leisure activities enhance social support, emotional wellbeing, and overall life satisfaction (Parra-Rizo et al., 2022). Maintaining these functions is the basis for dignity and social participation. These factors are not only indicators of health status, but also provide precise targets for preventive interventions (e.g., social promotion programs, exercise prescription) (Kris-Etherton et al., 2021).

Geriatric depression has a complex etiology and diverse manifestations. The Comprehensive Geriatric Assessment (CGA) is a multidimensional approach that assesses medical, psychological, and functional abilities (Schutz et al., 2022). It has been shown to be uniquely valuable for systematically identifying predictive features and individuals at high risk of geriatric depression. Multiple studies confirm that CGA dimensions such as functional dependence, cognitive impairment, polypharmacy, and social isolation significantly predict depression development in the elderly (Dafsari et al., 2023). Its strength lies in its ability to go beyond a single biological perspective and to capture the complex biopsychosocial interactions behind geriatric depression (Zhang et al., 2024).

Although CGA offers a rich database for identifying geriatric depression risks and multidimensional risk and protective factors are well recognized, there is no user-friendly, visual predictive tool based on CGA data. This gap limits CGA’s full potential for prospective prediction and personalized prevention. This gap limits the full potential of the CGA for prospective risk prediction and personalized prevention. To address this critical need, this study aims to construct an integrated depression screening model using the core components of the CGA. We hypothesized that lower levels of social support, greater functional impairment, and higher anxiety or pain scores—as measured by the CGA—would be significantly associated with depressive symptoms in elderly patients.

## 2 Methods

### 2.1 Study population, general characteristics, and chronic diseases

A cross-sectional design was selected to explore associations between CGA parameters and depressive symptoms in a defined hospital-based elderly population. The determination of the study sample size followed the rule that the number of events must be at least 10 times the number of variables in the model (Steyerberg, 2016). From June 2021 to June 2023, we conducted a cross-sectional study of 219 elderly patients from the Geriatrics Department of Fuzhou First General Hospital Affiliated with Fujian Medical University. All elderly patients aged ≥65 years with complete comprehensive geriatric assessment data who agreed to participate in the study were included. Patients unable to walk due to neuromusculoskeletal disorders, such as severe cerebrovascular disease, Parkinson’s disease, knee or hip osteoarthritis, and lumbar spinal stenosis, as well as participants with severe cognitive impairment, autoimmune dis-eases, severe liver and kidney dysfunction, or malignant tumors, were excluded from the study. The study was conducted in accordance with the Declaration of Helsinki and approved by The Ethics Committee of The Fuzhou First General Hospital Affiliated with Fujian Medical University (Approval Number: 20230304). A structured and pre-tested questionnaire was administered by trained clinicians to collect baseline sociodemographic and clinical data. Patient name, sex, age, BMI, hospitalization number, and chronic diseases (diabetes, cardiovascular disease, peptic ulcer disease) were evaluated using a custom-designed face-to-face interview questionnaire. Comorbidity data were initially collected from patient interviews and subsequently verified using electronic medical records, covering all records from 1 year before admission to 6 months after the first diagnosis. The medical record system used ICD-10 and ICD-9-CM codes to identify the comorbid conditions. Patients were randomly assigned using a computer-generated sequence in a 7: 3 ratio to either the training (*n* = 153) or validation (*n* = 66) cohort.

### 2.2 Comprehensive geriatric assessment

A comprehensive geriatric assessment (CGA) was performed by trained clinicians on the first day of admission for patients who met the inclusion criteria. The assessment mainly included the following: The 15-item Geriatric Depression Scale (GDS-15), which was culturally validated for Chinese populations (Zhang et al., 2020), is a short form of the GDS and is used to screen, diagnose, and evaluate depression in elderly individuals. Patients with a GDS-15 score ≥5 were considered to have clinically significant depressive symptoms, as per established thresholds in prior validation studies (Shin et al., 2019). When the critical score is <5 points indicates no depressive status, and ≥5 points indicates depressive status, the higher the score is, the more obvious the depression tendency. The Self-Assessment Scale for Anxiety (SAS) contains 20 items reflecting subjective feelings of anxiety, and each item is rated on a four-point scale according to the frequency of symptoms (Yue et al., 2020). The SAS was developed to assess anxiety: <50 points for no anxiety symptoms, 50–59 points for mild anxiety, 60–69 points for moderate anxiety and ≥69 points for severe anxiety. The Mini Nutritional Assessment (MNA) can be used to rapidly and effortlessly evaluate the nutritional status of geriatric patients in a reliable and noninvasive manner (Mastronuzzi and Grattagliano, 2019). The MNA score ranged from ≥24 points for good nutrition, 17–23.5 points for potentially poor nutrition and 0–17 points for poor nutrition. The SSRS (Social Support Rating Scale) was originally developed by Xiao Shuiyuan in 1986 for the Chinese population (Mcmahon et al., 2017). It has already been widely used in various studies in different Chinese communities and has been shown to have good validity and reliability, with scores ranging from 0 to 19 points for less social support, with scores ranging from 20 to 30 points for general social support and >30 points for satisfactory social support. The insomnia assessment included the use of the AIS scale: 0–3 points for no insomnia disorder, 4–6 points for suspected insomnia, and 7–24 points for insomnia. BADL (Basic Activity of Daily Living) were evaluated through six subscales: Bathing, Dressing, Toileting, Indoor mobility, Continence of defecation, and Eating (Zhang et al., 2021). A total of 100 points indicated normal daily life function, 61–99 points indicated mild functional impairment, 41–60 points indicated moderate functional impairment, and ≤40 points indicated severe functional impairment. The insomnia assessment included the use of the AIS (Athens Insomnia Scale): 0–3 points for no insomnia disorder, 4–6 points for suspected insomnia, and 7–24 points for insomnia. The visual analog method was used for pain assessment, with scores ranging from 0 for no pain, 1–3 for mild pain, 4–6 for moderate pain and 7–10 for severe pain.

### 2.3 Statistical analysis process

SPSS 25.0 was used for the analysis of the data. Categorical data are presented as frequencies and proportions, and the variables were compared using either the chi-square test or Fisher’s exact test in univariate analysis. Continuous data are expressed as medians and interquartile ranges, and the Mann–Whitney U test was used for comparisons of these variables. To obtain the most accurate results, variables with a *p* value less than 0.10 (Chen et al., 2024) in the univariate analysis were subjected to stepwise backward multivariate logistic regression analysis. A two-tailed *p* value of less than 0.05 was considered to indicate statistical significance (*p* < 0.05). The adjusted odds ratios (aORs) and 95% confidence intervals (CIs) for the predictive features were obtained by means of multivariate-adjusted binary logistic regression. R version 3.6.1^1^ and the “rms” and “ggplot2” packages were used to construct a nomogram. The significant predictive features identified in the multivariate analysis were selected for inclusion in the prediction nomogram. In the nomogram, each variable is assigned a distinct predicted score, and the total score is obtained by summing all the scores. The total predicted scores are then converted into predicted probabilities. The receiver operating characteristic (ROC) curve and the area under the curve (AUC) were calculated to evaluate the accuracy of the nomogram. Based on the cutoff points, the sensitivity and specificity were calculated. Internal validation was performed using 1,000 bootstrap resamples and Hosmer–Lemeshow test were performed to test the internal validation and stability of fit. In addition, calibration plots were generated, and decision curve analysis (DCA) was performed to quantify the performance ability and clinical utility of the model.

## 3 Results

### 3.1 Participant characteristics and predictive features

Table 1 presents the general characteristics of the 219 patients, including 61.2% women and 38.8% men. Many subjects were assessed for various factors, including Album, Mini Nutritional Assessment (MNA), Mini-Mental State Examination (MMSE), Anxiety, Cardiovascular Disease (CVD), Peptic Ulcer Disease (PUD), Diabetes, Pain, Insomnia, Constipation, Basic Activity of Daily Living (BADL), and Age.

**Table 1:** Characteristics of the subjects according to the prevalence of depression, *N* (%).

Variable	ALL *N* = 219	No depression, *N*%	Depression, *N*%	*p*. overall
Gender:				0.923
Female	85 (38.81%)	66 (39.29%)	19 (37.25%)	
Male	134 (61.19%)	102 (60.71%)	32 (62.75%)	
Age	87.00 [78.00; 91.00]	86.50 [78.00; 91.00]	88.00 [79.50; 92.00]	0.361
Alb	38.20 [35.25; 41.00]	38.55 [35.10; 41.10]	37.80 [35.65; 40.45]	0.782
BADL:				<0.001
No	59 (26.94%)	50 (29.76%)	9 (17.65%)	
Mild	118 (53.88%)	102 (60.71%)	16 (31.37%)	
Moderate	33 (15.07%)	13 (7.74%)	20 (39.22%)	
Severe	9 (4.11%)	3 (1.79%)	6 (11.76%)	
MNA	22.00 [19.00; 25.00]	23.00 [19.00; 26.00]	20.00 [16.00; 22.75]	<0.001
MMSE	24.00 [20.00; 27.00]	24.00 [20.00; 27.00]	24.00 [20.00; 26.00]	0.714
Anxiety:				0.001
No	132 (60.27%)	110 (65.48%)	22 (43.14%)	
Mild	55 (25.11%)	42 (25.00%)	13 (25.49%)	
Moderate	23 (10.50%)	13 (7.74%)	10 (19.61%)	
Severe	9 (4.11%)	3 (1.79%)	6 (11.76%)	
Pain:				0.014
No	149 (68.04%)	122 (72.62%)	27 (52.94%)	
Yes	70 (31.96%)	46 (27.38%)	24 (47.06%)	
CVD:				0.106
No	114 (52.05%)	93 (55.36%)	21 (41.18%)	
Yes	105 (47.95%)	75 (44.64%)	30 (58.82%)	
Diabetes:				0.788
No	143 (65.30%)	111 (66.07%)	32 (62.75%)	
Yes	76 (34.70%)	57 (33.93%)	19 (37.25%)	
PUD:				0.195
No	190 (86.76%)	149 (88.69%)	41 (80.39%)	
Yes	29 (13.24%)	19 (11.31%)	10 (19.61%)	
Insomnia	2.00 [0.00; 10.00]	1.50 [0.00; 7.25]	6.00 [0.00; 11.50]	0.024
SSRS	27.00 [21.00; 32.50]	28.00 [23.00; 34.00]	21.00 [15.50; 27.50]	<0.001
Constipation:				0.490
No	123 (56.16%)	97 (57.74%)	26 (50.98%)	
Yes	96 (43.84%)	71 (42.26%)	25 (49.02%)	

As shown in Table 2, univariate and multivariate logistic regression analyses using the AIC-based backward procedure identified several independent factors influencing the onset of depression in elderly patients. These factors included SSRS (OR = 0.909; CI: 0.852–0.964, *p* = 0.002), Pain (OR = 3.092; CI: 1.128–8.924, *p* = 0.031), Anxiety (OR = 2.061; CI: 1.228–3.562, *p* = 0.007), BADL (OR = 5.060; CI: 2.471–11.82, *p* = 0), and Gender (OR = 3.034; CI: 1.073–9.498, *p* = 0.044).

**Table 2:** Independent predictors of depression in elderly patients.

Variable	Univariate analysis			Multivariate analysis		
	OR	95% CI	*p* value	OR	95% CI	*p* value
Constipation	1.006	1.006 (0.471–2.117)	0.988			
SSRS	0.915	0.915 (0.868–0.959)	0	0.91	0.909 (0.852–0.964)	0.002
Insomnia	1.034	1.034 (0.977–1.093)	0.24			
PUD	1.677	1.677 (0.546–4.704)	0.338			
Diabetes	1.069	1.069 (0.482–2.302)	0.866			
CVD	1.997	1.997 (0.948–4.278)	0.07	2.002	2.002 (0.746–5.579)	0.172
Pain	2.05	2.05 (0.954–4.396)	0.064	3.092	3.092 (1.128–8.924)	0.031
Anxiety	2.104	2.104 (1.398–3.242)	0	2.061	2.061 (1.228–3.562)	0.007
MMSE	0.996	0.996 (0.924–1.08)	0.925			
MNA	0.886	0.886 (0.812–0.964)	0.006	0.941	0.941 (0.831–1.063)	0.331
BADL	4.431	4.431 (2.475–8.652)	0	5.06	5.060 (2.471–11.82)	0
Alb	0.995	0.995 (0.917–1.081)	0.905			
Age	1.023	1.023 (0.981–1.068)	0.299			
Gender	1.988	1.988 (0.917–4.545)	0.09	3.034	3.034 (1.073–9.498)	0.044

### 3.2 Nomogram construction and validation

**Figure 1:**
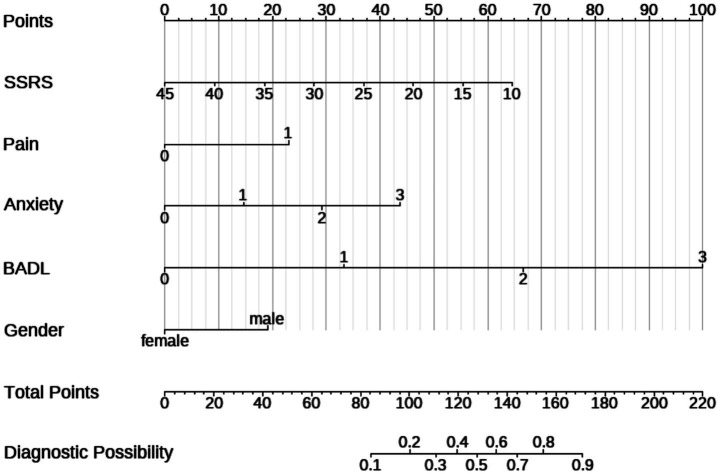
The nomogram model for the prediction of depression in elderly patients.

Utilizing these distinct variables, we created a nomogram to predict the risk of depression among senior patients, as illustrated in Figure 1. The location of each point is determined by the intersection of the respective variable with the vertical line of the point axis. By aggregating the points associated with each variable, we obtain the total risk score. The likelihood of depression can then be ascertained from the total score axis.

**Figure 2:**
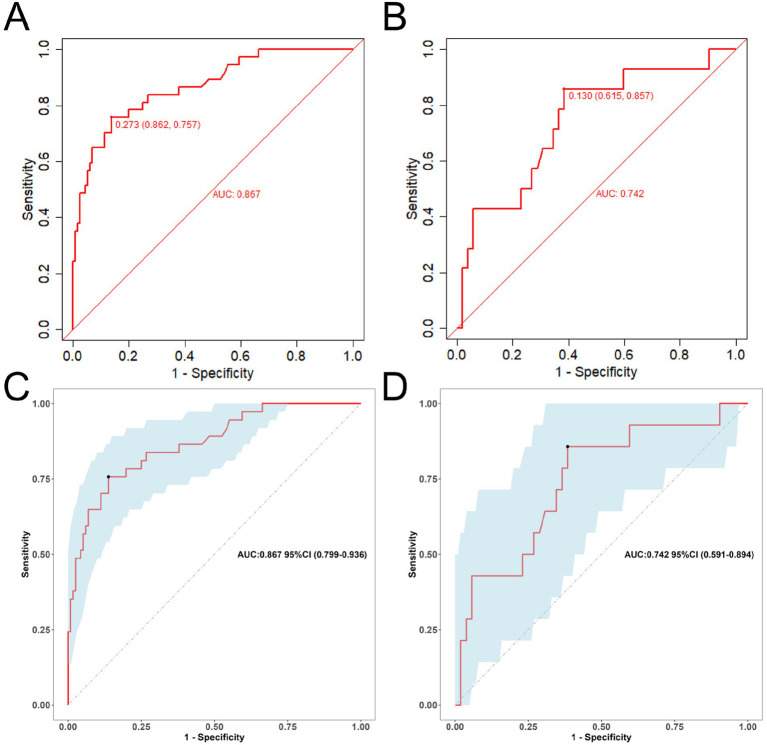
The ROC curve for assessing the predictive accuracy of the nomogram model (**A,C**: training set; **B,D**: test set).

We validated this nomogram. Collectively, these results indicate that the nomogram is a reliable tool for predicting depression in patients older than 65 years. The Hosmer–Lemeshow test for the training set was 0.172 (*p* > 0.05), suggesting good calibration. At the optimal cutoff value of 0.273 determined by maximizing Youden’s index, the model demonstrated a sensitivity of 0.862 and specificity of 0.757 in the training set (Figure 2A), and sensitivity of 0.615 and specificity of 0.857 in the test set (Figure 2B). The AUCs of the nomograms were 0.867 (95% CI: 0.799–0.936) in the training set (Figure 2C) and 0.724 (95% CI: 0.5919–0.894) in the test set, indicating robust discriminative ability in the training cohort and moderate generalizability in external validation.

**Figure 3:**
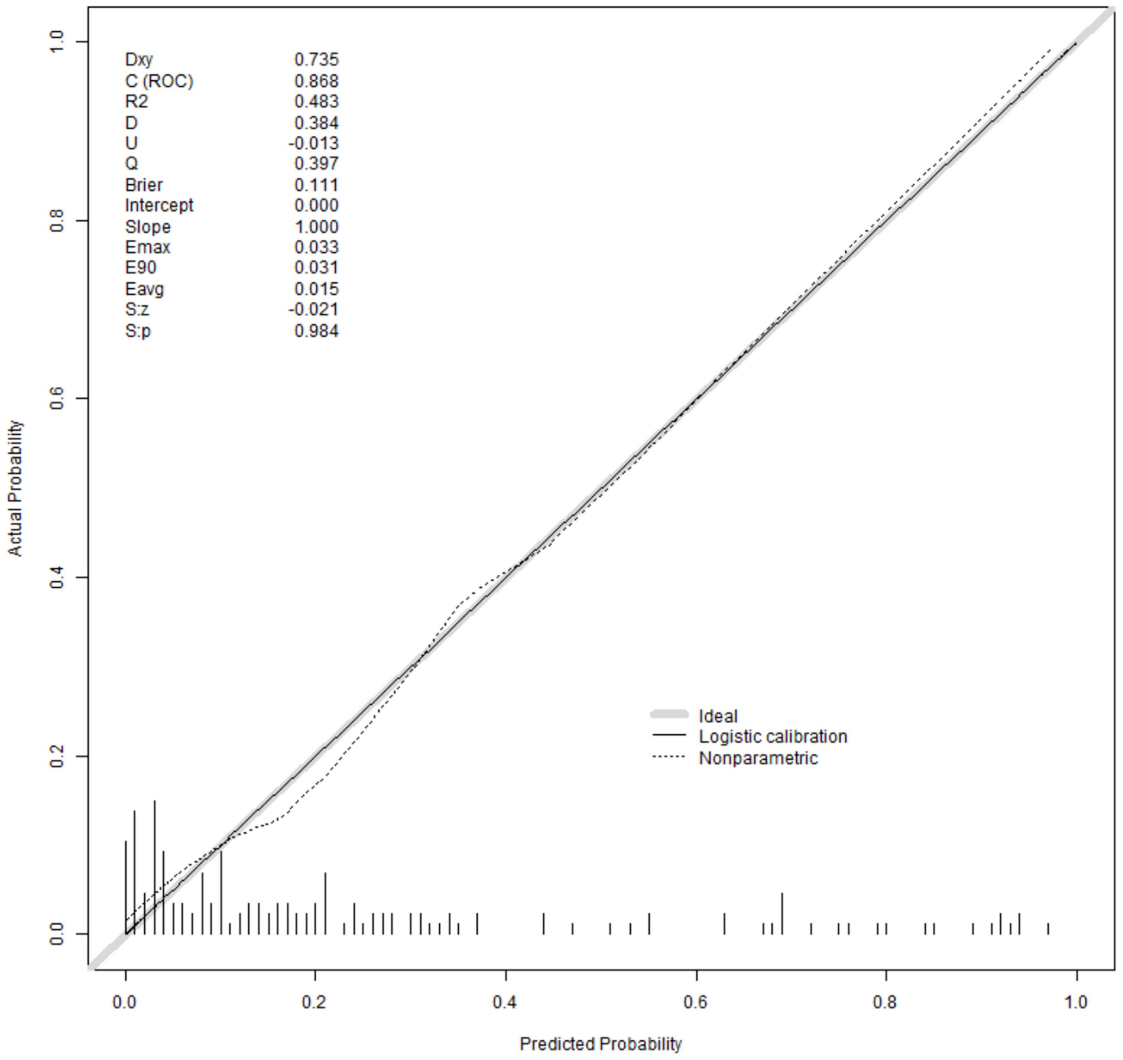
The calibration curve of the nomogram model.

**Figure 4:**
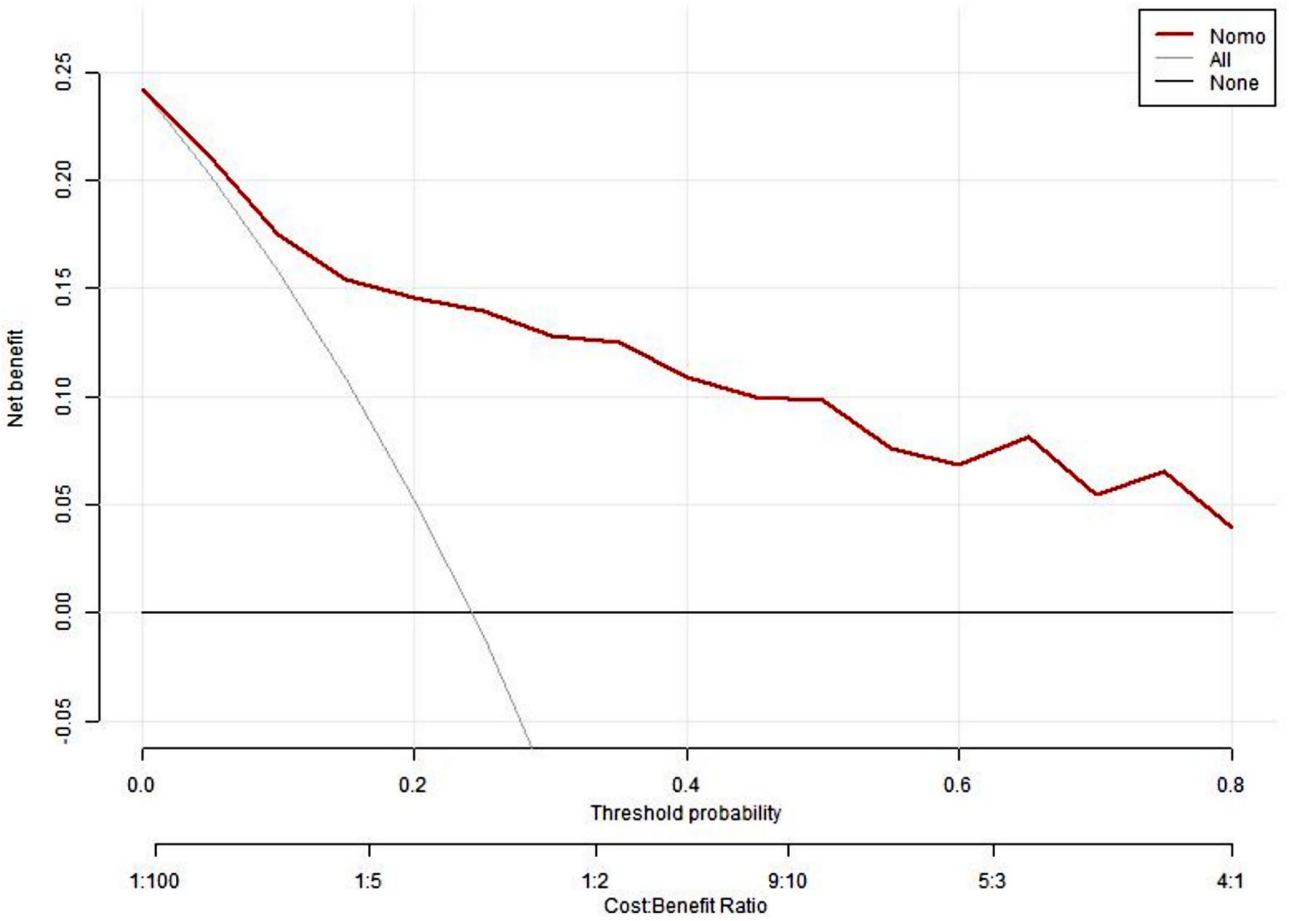
The DCA curve of the nomogram model.

Figure 3 presents the calibrated curve that was generated using the bootstrap method. This approach was iteratively employed 1,000 times to shape the curves, and the results revealed that both the bias-adjusted and visible curves closely mirrored the reference line, signifying a robust correlation between the estimated and actual risks of depression. A subsequent Hosmer–Lemeshow test of model adequacy yielded a chi-squared statistic of 5.265 with a *p* value of 0.729 reinforcing the adequacy of the model’s fit. Furthermore, to evaluate the clinical significance of our nomogram, we constructed a decision curve analysis (DCA) plot. The DCA curve findings indicated that the model provides a substantial net benefit across a substantial threshold range, ranging from approximately 5–80%, thereby underlining its practicality in guiding decision-making in clinical contexts (Figure 4).

## 4 Discussion

This study represents a novel approach for constructing a depression screening model of depression risk in elderly patients. The incorporation of CGA parameters represents a significant advance in the field. The findings of this study indicate that a number of indicators are strongly associated with depression. In particular, the results indicate that anxiety level, pain, level of social support (SSRS), ability to perform activities of daily living (BADL) and gender are associated with depression in elderly patients. The calibration curves of the modeling and verification groups demonstrated that the model exhibited favorable discrimination and fit. Depression is characterized by a low mood or feeling sad and a loss of interest or pleasure in daily activities.

In the relationship between depression and anxiety, previous studies have suggested that anxiety and depression are causative of each other, with similarities in biological basis and causative factors, and that they can produce pathological shaping of the patient’s personality, which is reversible but irreversible to the physiological functions of the organism (Frances et al., 1992). Although anxiety and depression are considered two distinct entities according to the diagnostic criteria, anxious depression is a relatively common syndrome (Choi et al., 2020). Research indicates that a broad range of depressive and anxiety symptoms are linked to a heightened risk of disability in the later stages of life (Dong et al., 2020).

A study revealed that among Chinese senior citizens, those experiencing difficulties in basic and instrumental activities of daily living (BADL/IADL), particularly BADL, had a significant correlation with depressive symptoms (Liu et al., 2023). The dynamics of this relationship are complex. Seniors often face initial issues with instrumental activities of daily living (IADL), progressing to issues with basic activities of daily living (BADL). These issues lead to decreased purposeful actions, negative affect, and emotional impacts from physical decline. Impairments can disrupt routines and social interaction and can trigger depression (Bacon et al., 2016; Bruce, 2001; Luo et al., 2017). Moreover, these impairments can curtail daily routines and diminish social interaction, contributing to the onset of depression (Bacon et al., 2016; Bruce, 2001).

Chronic pain demonstrates significant predictive value for depression onset and progression in elderly populations, with pain-related functional interference emerging as the strongest independent predictor (Landi et al., 2005). This suggests that chronic pain may not only directly affect mood through biological pathways involving cytokines and neuroinflammation, but also indirectly by impairing physical function and reducing quality of life. Prospective investigations reveal a dose–response relationship, evidenced by a Hong Kong cohort study (*N* = 318) where baseline pain severity predicted depressive symptom exacerbation at 12-month follow-up (*β* = 0.32, *p* < 0.01) after adjusting for confounding factors (Chou and Chi, 2005). This temporal association is further substantiated by large-scale data (*N* = 5,372) showing daily pain exposure correlates with 44% higher depression scores (3.6 vs. 2.5 points) and 1.5–2.3 fold increased risks of core depressive symptoms compared to pain-free individuals (Landi et al., 2005).

Gender serves as a significant predictor of depression in elderly patients, with robust evidence highlighting sex-specific disparities in prevalence and clinical manifestations. These findings suggest not only biological (e.g., hormonal) but also psychosocial determinants—such as caregiving roles, social expectations, and access to care—may explain gender disparities in late-life depression. Population-based studies reveal that elderly women exhibit higher rates of depressive symptoms compared to men, with community-dwelling women showing a 15% prevalence of depressive symptoms versus 1–3% for major depressive disorder in men (Heo et al., 2023). This disparity persists in disease-specific cohorts: women with heart failure demonstrated nearly double the prevalence of clinically significant depressive symptoms (CES-D ≥ 16: 39% vs. 21%, *p* < 0.001) (Heo et al., 2023), and those with chronic vestibular dysfunction showed stronger associations between female sex and depressive symptom severity (*β* = 1.32, *p* < 0.05) (Gazzola et al., 2009). Metabolic syndromestudies further revealed sex-dimorphic patterns, where male patients with optic neuritis exhibited a significantly higher risk of developing depressive disorders compared to female patients, with an adjusted hazard ratio (HR) of 1.450 (95% CI: 1.338–1.571) (Kim et al., 2025). These findings underscore sex-related biological vulnerabilities sociocultural factors as key mediators of depression risk stratification by gender in aging populations (Targum et al., 1992).

More importantly, however, we found a significant association between depression and the Mini Nutritional Assessment score, which has previously been shown to reflect nutritional levels in elderly hospitalized patients. Malnutrition may contribute to depression through mechanisms involving reduced tryptophan availability, impaired neurotransmitter synthesis, and inflammation. As the world’s population continues to age, researchers in a variety of fields are becoming increasingly concerned about the intersection of nutrition and depression in elderly people. A pertinent study published in the last year’s issue of nutrition highlighted a profound link between nutritional status and depression (Mcfarland et al., 2020). Similar research has also shown that elderly patients suffering from depression tend to score significantly lower on the Mini Nutritional Assessment than those without depression, suggesting a possible link between depression and malnutrition (German et al., 2008).

Our study revealed that higher levels of social support, which refers to the unpaid help that vulnerable groups in society receive from outside sources, such as moral and material assistance and services, significantly reduced the risk of depression in older patients. It is plausible that social support mitigates the psychological burden of functional limitations and chronic pain, thereby attenuating the depressive response. Consistent with our study, our research revealed that social support can affect an individual’s cognitive functioning and behavior and has a greater impact on the mental health of older people (Muhammad and Maurya, 2022). In particular, during the COVID-19 pandemic in recent years, social isolation has had a serious impact on the physical and mental health of older people, including mental illnesses such as anxiety and depression (Sepúlveda-Loyola et al., 2020). Another study showed that with strong social support, depressive symptoms were significantly reduced, and even chronic pain was reduced with increased levels of support (Sepúlveda-Loyola et al., 2020; Hung et al., 2017). Family support, which focuses on emotional wellbeing, such as respect and under-standing, aids in reducing depression among elderly people. However, the importance of social support from friends is equally crucial and is becoming a growing area of focus (Taylor et al., 2018).

The present study has the following limitations. First, this model identifies variables statistically associated with depression status but cannot infer causality due to the cross-sectional design. Features such as anxiety may be consequences rather than causes of depression. Future longitudinal studies are required to establish causal roles. Second, the samples included were all elderly hospitalized patients aged 65 years and above, which cannot represent the entire elderly population. Since participants were all hospitalized, they may exhibit higher baseline functional impairment and comorbidity burden, which may overestimate the prevalence of depressive symptoms compared to community-dwelling older adults. In addition, the integrity and credibility of the comprehensive geriatric assessment are compromised by differences in medical standards between healthcare institutions and in patients’ understanding of their own past health, which affects the assessment results. Although our comprehensive geriatric assessment comprehensively assesses the overall condition of elderly people, some scales related to nutritional status and frailty, such as gait speed, grip strength, and leg circumference, were not included in this experiment. Future research on older people will continue to improve this topic.

This model has shown potential in aiding healthcare professionals with the early detection of patients at high risk and the formulation of tailored intervention strategies. Considering that machine learning methods can capture complex nonlinear relationships and interactions, future studies will further investigate the application of methods such as Random Forests, Gradient Boosting Machines (XGBoost, LightGBM), Support Vector Machines (SVM), and Neural Networks (Du et al., 2025a; Du et al., 2025b). To confirm its extensive applicability, especially in light of the constraints imposed by a single-center data source and a moderate sample size, it is essential to perform external validation across multicenter cohorts and assess its predictive accuracy over time.

## 5 Conclusion

Geriatric depression is closely related to a variety of predictive features (i.e., multiple geriatric syndromes); thus, healthcare professionals should not only focus on the surface of geriatric depression but also intervene in geriatric syndromes. Second, the depression probability estimator constructed in this study is also effective, and early screening of predictive features will help healthcare professionals provide timely guidance to patients to prevent the occurrence of geriatric depression. Second, the model demonstrated robust performance in our study and may constitute a valuable tool for clinical screening, contingent upon external validation.

## Data Availability

The raw data supporting the conclusions of this article will be made available by the authors without undue reservation.

## Footnotes

^1^ http://www.r-project.org

## References

[ref1] Bacon K. L. Heeren T. Keysor J. J. Stuver S. O. Cauley J. A. Fredman L. (2016). Longitudinal and reciprocal relationships between depression and disability in older women caregivers and noncaregivers. The Gerontologist 56, 723–732. doi: 10.1093/geront/gnu157, PMID: 26035874 PMC4944533

[ref3] Borges M. K. Jeuring H. W. Marijnissen R. M. van Munster B. C. Aprahamian I. van den Brink R. H. S.. (2022). Frailty and affective disorders throughout adult life: a 5-year follow-up of the lifelines cohort study. J. Am. Geriatr. Soc. 70, 3424–3435. doi: 10.1111/jgs.18021, PMID: 36054011 PMC10086828

[ref4] Borges M. K. Romanini C. V. Lima N. A. Petrella M. da Costa D. L. An V. N.. (2021). Longitudinal association between late-life depression (LLD) and frailty: findings from a prospective cohort study (MiMiCS-FRAIL). J. Nutr. Health Aging 25, 895–902. doi: 10.1007/s12603-021-1639-x, PMID: 34409968 PMC8103429

[ref5] Bruce M. L. (2001). Depression and disability in late life: directions for future research. Am. J. Geriatr. Psychiatry 9, 102–112. doi: 10.1097/00019442-200105000-00003, PMID: 11316615

[ref6] Chen Y. Jiang J. He M. Zhong K. Tang S. Deng M.. (2024). Nomogram for predicting difficult total laparoscopic hysterectomy: a multi-institutional, retrospective model development and validation study. Int. J. Surg. 110, 3249–3257. doi: 10.1097/JS9.0000000000001406, PMID: 38537077 PMC11175783

[ref7] Choi K. W. Kim Y. K. Jeon H. J. (2020). Comorbid anxiety and depression: clinical and conceptual consideration and transdiagnostic treatment. Adv. Exp. Med. Biol. 1191, 219–235. doi: 10.1007/978-981-32-9705-0_14, PMID: 32002932

[ref8] Chou K. L. Chi I. (2005). Reciprocal relationship between pain and depression in elderly Chinese primary care patients. Int. J. Geriatr. Psychiatry 20, 945–952. doi: 10.1002/gps.1383, PMID: 16163745

[ref9] Cole S. A. Sannidhi D. Jadotte Y. T. Rozanski A. (2023). Using motivational interviewing and brief action planning for adopting and maintaining positive health behaviors. Prog. Cardiovasc. Dis. 77, 86–94. doi: 10.1016/j.pcad.2023.02.003, PMID: 36842453

[ref10] Dafsari F. S. Bewernick B. Böhringer S. Domschke K. Elsaesser M. Löbner M.. (2023). Cognitive behavioral therapy for late-life depression (CBTlate): results of a Multicenter, randomized, observer-blinded, controlled trial. Psychother. Psychosom. 92, 180–192. doi: 10.1159/0005294, PMID: 37004508

[ref11] Dai X. Liu S. Li X. Chen K. Gao S. Wang J.. (2024). Longitudinal association between depressive symptoms and cognitive function: the neurological mechanism of psychological and physical disturbances on memory. Psychol. Med. 54, 3602–3611. doi: 10.1017/S0033291724001612. Epub ahead of print, PMID: 39397683 PMC11536121

[ref12] Dong L. Freedman V. A. Mendes De Leon C. F. (2020). The association of comorbid depression and anxiety symptoms with disability onset in older adults. Psychosom. Med. 82, 158–164. doi: 10.1097/PSY.0000000000000763, PMID: 31688675 PMC7007837

[ref13] Du J. Tao X. Zhu L. Qi W. Min X. Deng H.. (2025a). A risk prediction system for depression in middle-aged and older adults grounded in machine learning and visualization technology: a cohort study. Front. Public Health 13:1606316. doi: 10.3389/fpubh.2025.1606316, PMID: 40535435 PMC12173875

[ref14] Du J. Tao X. Zhu L. Wang H. Qi W. Min X.. (2025b). Development of a visualized risk prediction system for sarcopenia in older adults using machine learning: a cohort study based on CHARLS. Front. Public Health 13:1544894. doi: 10.3389/fpubh.2025.1544894, PMID: 40144970 PMC11936879

[ref15] Frances A. Manning D. Marin D. Kocsis J. McKinney K. Hall W.. (1992). Relationship of anxiety and depression. Psychopharmacology 106, S82–S86. doi: 10.1007/BF02246243, PMID: 1546149

[ref16] Gao Y. Zhang Z. Song J. Gan T. Lin Y. Hu M.. (2024). Combined healthy lifestyle behaviours and incident dementia: a systematic review and dose-response meta-analysis of cohort studies. Int. J. Nurs. Stud. 156:104781. doi: 10.1016/j.ijnurstu.2024.104781, PMID: 38744152

[ref17] Gazzola J. M. Aratani M. C. Doná F. Macedo C. Fukujima M. M. Ganança M. M.. (2009). Factors relating to depressive symptoms among elderly people with chronic vestibular dysfunction. Arq. Neuropsiquiatr. 67, 416–422. doi: 10.1590/s0004-282x2009000300009, PMID: 19623437

[ref18] GermanL.FeldblumI.BilenkoN.CastelH.Harman-BoehmI.ShaharD. R. (2008). Depressive symptoms and risk for malnutrition among hospitalized older people. J. Nutr. Health Aging 12, 313–318. doi: 10.1007/BF0298266118443713

[ref19] Gundersen E. Bensadon B. (2023). Geriatric depression. Prim. Care 50, 143–158. doi: 10.1016/j.pop.2022.10.010, PMID: 36822724

[ref20] Han J. W. Yang H. W. Bae J. B. Oh D. J. Moon D. G. Lim E.. (2023). Shared risk factors for depressive disorder among older adult couples in Korea. JAMA Netw. Open 6:e238263. doi: 10.1001/jamanetworkopen.2023.8263, PMID: 37058304 PMC10105310

[ref21] Heo S. Shin M. S. Lee M. O. Kim S. Kim S. H. Randolph J.. (2023). Factors related to patients' self-care and self-care confidence in Korean patients with heart failure and their caregivers: a cross-sectional, correlational study. J. Cardiovasc. Nurs. 38, 140–149. doi: 10.1097/JCN.0000000000000922, PMID: 35507026

[ref22] Hung M. Bounsanga J. Voss M. W. Crum A. B. Chen W. Birmingham W. C. (2017). The relationship between family support; pain and depression in older people with arthritis. Psychol. Health Med. 22, 75–86. doi: 10.1080/13548506.2016.1211293, PMID: 27427504

[ref9001] Kim J. Jang S. Choi J. Han K. Jung J. H. Oh S. Y.. (2025). Association of optic neuritis with incident depressive disorder risk in a Korean nationwide cohort. Sci Rep 15, 7764. doi: 10.1038/s41598-025-92370-5, PMID: 40044803 PMC11882889

[ref23] Kris-Etherton P. M. Petersen K. S. Després J. P. Braun L. de Ferranti S. D. Furie K. L.. (2021). Special considerations for healthy lifestyle promotion across the life span in clinical settings: a science advisory from the American Heart Association. Circulation 144, e515–e532. doi: 10.1161/CIR.0000000000001014, PMID: 34689570

[ref24] Landi F. Onder G. Cesari M. Russo A. Barillaro C. Bernabei R.. (2005). Pain and its relation to depressive symptoms in frail older people living in the community: an observational study. J. Pain Symptom Manag. 29, 255–262. doi: 10.1016/j.jpainsymman.2004.06.016, PMID: 15781176

[ref25] Li H. Liu X. Zheng Q. Zeng S. Luo X. (2022). Gender differences and determinants of late-life depression in China: a cross-sectional study based on CHARLS. J. Affect. Disord. 309, 178–185. doi: 10.1016/j.jad.2022.04.059, PMID: 35472476

[ref26] Linnan L. A. Vaughn A. E. Smith F. T. Westgate P. Hales D. Arandia G.. (2020). Results of caring and reaching for health (CARE): a cluster-randomized controlled trial assessing a worksite wellness intervention for child care staff. Int. J. Behav. Nutr. Phys. Act. 17:64. doi: 10.1186/s12966-020-00968-x, PMID: 32414381 PMC7227251

[ref27] Liu H. Ma Y. Lin L. Sun Z. Li Z. Jiang X. (2023). Association between activities of daily living and depressive symptoms among older adults in China: evidence from the CHARLS. Front. Public Health 11:1249208. doi: 10.3389/fpubh.2023.1249208, PMID: 38035294 PMC10687586

[ref28] Loyal M. S. Numbers K. Reppermund S. Brodaty H. Sachdev P. S. Mewton L.. (2025). Longitudinal associations between late-life depression, cerebrovascular disease and cognition. J. Affect. Disord. 376, 59–67. doi: 10.1016/j.jad.2025.01.147, PMID: 39892757

[ref29] Luo Y. N. Wang Z. J. Zheng X. Y. (2017). Association between the change of daily living activities and symptoms of depression in Chinese middle-aged and older people. Zhonghua Liu Xing Bing Xue Za Zhi = Zhonghua Liuxingbingxue Zazhi 38, 1055–1059. doi: 10.3760/cma.j.issn.0254-6450.2017.08.011, PMID: 28847053

[ref30] Mastronuzzi T. Grattagliano I. (2019). Nutrition as a health determinant in older people patients. Curr. Med. Chem. 26, 3652–3661. doi: 10.2174/0929867324666170523125806 28545376

[ref31] Mcfarland D. C. Breitbart W. Miller A. H. Nelson C.. (2020). Depression and inflammation in patients with lung cancer: a comparative analysis of acute phase reactant inflammatory markers. Psychosomatics 61, 527–537. doi: 10.1016/j.psym.2020.03.005, PMID: 32331769 PMC7529727

[ref32] Mcmahon E. M. Corcoran P. O’regan G. Keeley H. Cannon M. Carli V.. (2017). Physical activity in European adolescents and associations with anxiety, depression and well-being. Eur. Child Adolesc. Psychiatry 26, 111–122. doi: 10.1007/s00787-016-0875-9, PMID: 27277894

[ref33] Muhammad T. Maurya P. (2022). Social support moderates the association of functional difficulty with major depression among community-dwelling older adults: evidence from LASI, 2017–18. BMC Psychiatry 22:317. doi: 10.1186/s12888-022-03959-3, PMID: 35509005 PMC9066756

[ref34] Parra-Rizo M. A. Vásquez-Gómez J. Álvarez C. Diaz-Martínez X. Troncoso C. Leiva-Ordoñez A. M.. (2022). Predictors of the level of physical activity in physically active older people. Behav. Sci. (Basel) 12:331. doi: 10.3390/bs12090331, PMID: 36135135 PMC9495331

[ref35] Schutz N. Botros A. Hassen S. B. Saner H. Buluschek P. Urwyler P.. (2022). A sensor-driven visit detection system in older adults' homes: towards digital late-life depression marker extraction. IEEE J. Biomed. Health Inform. 26, 1560–1569. doi: 10.1109/JBHI.2021.3114595, PMID: 34550895

[ref36] Sepúlveda-Loyola W. Rodríguez-Sánchez I. Pérez-Rodríguez P. Ganz F. Torralba R. Oliveira D. V.. (2020). Impact of social isolation due to COVID-19 on health in older people: mental and physical effects and recommendations. J. Nutr. Health Aging 24, 938–947. doi: 10.1007/s12603-020-1469-2 33155618 PMC7597423

[ref37] Shin C. Park M. H. Lee S. H. Ko Y. H. Kim Y. K. Han K. M.. (2019). Usefulness of the 15-item geriatric depression scale (GDS-15) for classifying minor and major depressive disorders among community-dwelling elders. J. Affect. Disord. 259, 370–375. doi: 10.1016/j.jad.2019.08.053, PMID: 31470180

[ref38] Steyerberg E. W. (2016). Regression modeling strategies: with applications, to linear models, logistic and ordinal regression, and survival analysis. Biometrics 72, 1006–1007. doi: 10.1111/biom.12569

[ref39] Targum S. D. Marshall L. E. Fischman P. (1992). Variability of TRH test responses in depressed and normal elderly subjects. Biol. Psychiatry 31, 787–793. doi: 10.1016/0006-3223(92)90310-v, PMID: 1643193

[ref40] Taylor H. O. Taylor R. J. Nguyen A. W. Chatters L. (2018). Social isolation, depression, and psychological distress among older adults. J. Aging Health 30, 229–246. doi: 10.1177/0898264316673511, PMID: 28553785 PMC5449253

[ref41] Wang B. Lan C. Liu K. Fu L. Zhang P. Ao C.. (2025). Global, regional, and national burden and attributable risk factors of depressive disorders among older adults, 1990-2021. Int. Psychogeriatr. 4:100069. doi: 10.1016/j.inpsyc.2025.100069, PMID: 40187958

[ref42] Wang Z. Yang H. Zheng P. Liu B. Guo Z. Geng S.. (2020). Life negative events and depressive symptoms: the China longitudinal ageing social survey. BMC Public Health 20:968. doi: 10.1186/s12889-020-09119-0, PMID: 32560710 PMC7305594

[ref43] Xue L. Lewis E. Bocharova M. Young A. H. Aarsland D. (2025). Decreased neutrophil-to-lymphocyte ratio predicted cognitive improvement in late-life depression treated with vortioxetine: findings from an eight-week randomized controlled trial. Brain Behav. Immun. 126, 53–58. doi: 10.1016/j.bbi.2025.01.029, PMID: 39921151

[ref44] Yue T. Li Q. Wang R. Liu Z. Guo M. Bai F.. (2020). Comparison of hospital anxiety and depression scale (HADS) and Zung self-rating anxiety/depression scale (SAS/SDS) in evaluating anxiety and depression in patients with psoriatic arthritis. Dermatology (Basel, Switzerland) 236, 170–178. doi: 10.1159/000498848, PMID: 31434087

[ref45] Zhang H. Wang S. Wang L. Yi X. Jia X. Jia C. (2020). Comparison of the geriatric depression scale-15 and the patient health questionnaire-9 for screening depression in older adults. Geriatr Gerontol Int 20, 138–143. doi: 10.1111/ggi.13840, PMID: 31820572

[ref46] Zhang L. Wang L. Yu M. Wu R. Steffens D. C. Potter G. G.. (2024). Hybrid representation learning for cognitive diagnosis in late-life depression over 5 years with structural MRI. Med. Image Anal. 94:103135. doi: 10.1016/j.media.2024.103135, PMID: 38461654 PMC11016377

[ref47] Zhang Y. Xiong Y. Yu Q. Shen S. Chen L. Lei X.. (2021). The activity of daily living (ADL) subgroups and health impairment among Chinese elderly: a latent profile analysis. BMC Geriatr. 21:30. doi: 10.1186/s12877-020-01986-x, PMID: 33413125 PMC7791986

